# Study on the anisotropic characteristics of mechanical properties and energy evolution in layered phyllite

**DOI:** 10.1371/journal.pone.0341889

**Published:** 2026-02-12

**Authors:** Luhai Chen, Baoping Xi, Na Zhao, Yunsheng Dong, Shuixin He, Keliu Liu, Pengli Gao, Jin Xie

**Affiliations:** 1 College of Mining Engineering, Taiyuan University of Technology, Taiyuan, Shanxi, China; 2 Key Laboratory of In-situ Property-improving Mining of Ministry of Education, Taiyuan University of Technology, Taiyuan, Shanxi, China; 3 College of Safety and Emergency Management, Shanxi Vocational University of Engineering Science and Technology, Taiyuan, Shanxi, China; China Construction Fourth Engineering Division Corp. Ltd, CHINA

## Abstract

To investigate the anisotropic characteristics of the mechanical properties and energy evolution in layered rock masses, conventional triaxial compression tests were carried out on layered phyllite specimens with different bedding angles (0°, 30°, 45°, 60°, and 90°). Based on the test results and the law of energy conservation, the anisotropic mechanical behavior and energy evolution were analyzed. The results show that the deformation characteristics of layered phyllite under different confining pressures and bedding angles are similar, with overall brittle failure. The bedding angle and confining pressure significantly affect its dilation behavior. The peak strength, elastic modulus, internal friction angle, and cohesion of the layered phyllite exhibit a “U”-shaped distribution, first decreasing and then increasing, with significant anisotropy. However, the anisotropy of the Poisson’s ratio is not pronounced. The increase in confining pressure enhances the mechanical properties of the layered phyllite and reduces its anisotropic behavior. The failure modes of the layered phyllite primarily include shear failure along the bedding planes and tensile splitting failure along the bedding planes, both showing significant bedding and confining pressure effects. The energy evolution of layered phyllite with different bedding angles is similar. The peak total absorption energy (*U*_p_), peak elastic strain energy (*U*_p_^e^), and peak dissipated energy (*U*_p_^d^) first decrease and then increase as the bedding angle increases. Additionally, these energies exhibit a nonlinear increase with increasing confining pressure.

## Introduction

Layered rocks, as a standard geological structure, are widely present in sedimentary rocks, metamorphic rocks, and certain igneous rocks [1–6]. Their significant bedding structure makes their physical and mechanical properties (such as strength, deformation, permeability, fracture evolution, and failure modes) strongly influenced by weak planes (i.e., bedding planes, stratification, fissures, and joints), exhibiting apparent anisotropy [7–15]. This anisotropic behavior significantly impacts the design and stability of rock engineering. Therefore, a thorough study of layered rocks’ anisotropic characteristics is fundamental for rock engineering design and crucial for ensuring engineering safety and improving operational efficiency.

In the past few decades, scholars have conducted extensive research on the anisotropic properties of layered rocks through experimental methods, numerical simulations, and theoretical analysis. Studies have shown that the strength, elastic modulus, and failure modes of layered rocks depend on the bedding angle and loading conditions [15–20]. Behrestaghi et al. [21] noted that schist’s deformation modulus and triaxial compressive strength exhibit a “U”-shaped variation with bedding angle β. As confining pressure increases, cohesion continuously increases while the friction angle decreases. The maximum and minimum values of cohesion occur at *β* = 90° and *β* = 30°–45°, respectively. McCabe and Koerner [22] found that the bedding angle *β* significantly affects the shear strength and compressive strength of mica schist under compression, with the maximum values of shear strength and friction angle occurring at β < 30° and β > 70°, and the minimum values occurring at *β *= 50°–59°. Chen et al. [23] studied the anisotropic mechanical properties of phyllite through conventional triaxial tests, finding a “U”-shaped relationship between peak strength and bedding angle, with the anisotropic mechanical properties weakening as confining pressure increases. Liu et al. [24] conducted experiments and numerical simulations to investigate the influence of bedding plane characteristics on layered rocks’ mechanical properties and micro-damage behavior. They found that the strength and roughness of bedding planes significantly affect the rock mass’s uniaxial compressive strength and failure modes.

Rock failure involves energy input, accumulation of elastic strain energy, energy dissipation, and energy release [25,26]. The accumulation and release of energy are prerequisites for macroscopic rock mass failure, particularly in layered rocks. Due to the presence of anisotropic characteristics, energy distribution and transformation in layered rocks exhibit clear directional dependence [27–31]. Currently, research on the energy evolution laws of rock deformation and failure processes mainly focuses on three aspects: experimental studies on energy evolution processes and characteristics, constitutive models, criteria, indicators based on energy principles, and numerical simulations of energy evolution. Wang et al. [32] investigated the mechanical properties and energy evolution process of granite through experiments. They proposed that the Mogi-Coulomb strength criterion better describes the unloading failure strength of rocks. Zhang et al. [33] established a new indicator to evaluate coal brittleness from the perspective of energy evolution. Zhang et al. [34] conducted triaxial cyclic loading and unloading tests on sandstone, analyzing the evolution laws and fractal characteristics of elastic, dissipated, and input energy during the loading and unloading processes. Chen et al. [35] performed uniaxial compression cyclic loading tests on jointed rock masses using the discrete element method based on laboratory experiments, revealing the macroscopic mechanical response and microscopic damage evolution characteristics under rock fatigue loading. However, most of the aforementioned research focuses on the anisotropic characteristics of rocks and constitutive models. At the same time, there is relatively little research on energy transformation in layered rocks’ deformation and failure processes. However, energy accumulation and release are typical features in the deformation process of layered rocks, and the energy evolution characteristics are closely related to the failure mechanisms of rocks. Therefore, further research on the energy evolution characteristics during layered rocks’ deformation and failure processes is of great significance.

The anisotropy of metamorphic rocks (whether inherent anisotropy or transverse isotropy) is generally higher than that of other types of rocks [21,36,37]. Phyllite, as a typical layered metamorphic rock, also exhibits significant anisotropic features in its physical and mechanical properties as well as in its energy evolution [5,38,39]. With the development of energy extraction, underground engineering, and geological disaster prediction, phyllite, as a typical representative of layered rock masses, has become an important research subject for exploring the mechanical properties and energy evolution laws of layered rocks.

Metamorphic rocks’ anisotropy (inherent anisotropy or transverse isotropy) is generally higher than other rocks [21,36,37]. Phyllite, a typical layered metamorphic rock, exhibits significant anisotropic features in its physical and mechanical properties and energy evolution [5,38,39]. With the development of energy extraction, underground engineering, and geological disaster prediction, phyllite, a typical representative of layered rock masses, has become an important research subject for exploring layered rocks’ mechanical properties and energy evolution laws.

This study used layered phyllite from the Shenzuo Tunnel of the Jiuma Expressway as the research object. Conventional triaxial compression tests were conducted on layered phyllite with different bedding angles (0°, 30°, 45°, 60°, and 90°). Based on the test results and the law of energy conservation, the anisotropic characteristics of the layered phyllite were studied, including its mechanical behavior, deformation parameters, failure modes, and energy evolution characteristics. The results of this study provide important theoretical foundations for the engineering design and disaster prevention of layered rock masses.

## Materials and methods

### Materials and sample preparation

**Fig 1:**
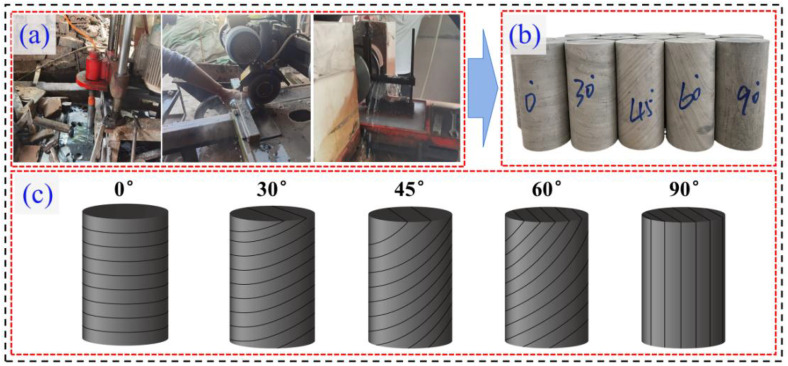
Preparation process of phyllite specimens (a) Core drilling, stone cutting, and grinding process (b) Final prepared standard cylindrical specimens (φ50 mm×100 mm) with different bedding angles (c) Schematic diagram of phyllite specimens with different bedding angles.

The layered phyllite samples used in the experiments were all collected from the same rock layer at adjacent locations along the same direction within the Shenzuo Tunnel of the Jiuma Expressway in Sichuan Province in order to minimize the variability of the layered phyllite samples. According to the International Society for Rock Mechanics and Rock Engineering (ISRM) test standards [40], the samples were processed using a core drilling machine, rock cutting machine, and end-face grinding machine into standard cylindrical specimens with a diameter of 50 mm and a height of 100 mm, possessing specific bedding angles (*β* = 0°, 30°, 45°, 60°, and 90°). The specimens’ surfaces were smooth and even, with no apparent defects. Based on this, a non-metallic ultrasonic tester was used to perform acoustic wave testing on the specimens, further selecting those with better uniformity. There were at least three specimens for each bedding angle. The process of preparing the phyllite specimens is shown in Fig 1.

### Experimental equipment and procedure

**Fig 2:**
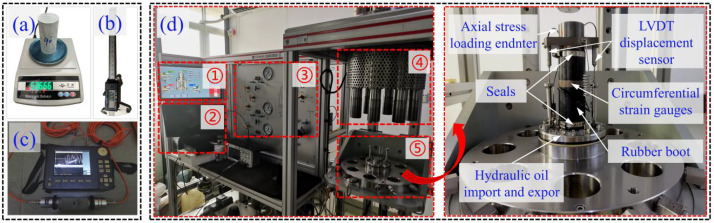
Experimental apparatus: (a) Electronic scale (b) Vernier caliper (c) ZBL-U520 non-metallic ultrasonic detector (d) ROCK 600−50 rock full-stress multi-field coupling triaxial testing apparatus ① Main system control display panel ② Data acquisition system ③ System valve operation panel ④ Axial pressure system ⑤ Triaxial pressure chamber.

Before conducting the triaxial compression tests, the mass, dimensions (height and diameter), and P-wave velocity of the phyllite specimens were measured using an electronic scale (Fig 2(a)), a vernier caliper (Fig 2(b)), and a ZBL-U520 non-metallic ultrasonic detector (Fig 2(c)). The prepared phyllite specimens were then subjected to conventional triaxial compression tests using the ROCK 600−50 rock full-stress multi-field coupling triaxial testing apparatus. The confining pressure was designed for 10 MPa, 20 MPa, and 30 MPa. During the confining pressure loading process, a stress control method was applied, with a 5 MPa/min loading rate to reach the design values, which were then held constant. Subsequently, axial loading was applied using a displacement control method, with a rate of 0.001 mm/s, until specimen failure occurred, as shown in Fig 2(d).

## Experimental results

### Physical properties of layered phyllite

#### Microstructure characterization.

**Fig 3:**
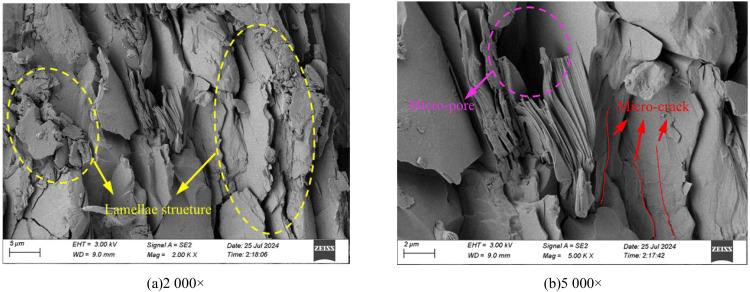
SEM images of phyllite specimens. (a) 2000× (b) 5000×.

The macroscopic mechanical properties of rocks are determined by their microstructure, and variations in the microstructure can effectively explain changes in their macroscopic mechanical behavior [41–43]. In this study, the microstructure of phyllite samples was analyzed using a scanning electron microscope (Zeiss Sigma 300), with magnifications set at 2000× and 5000×. Fig 3 shows the microstructural characteristics of the phyllite samples at different magnifications. As seen in the figure, the microstructure of phyllite is characterized by an apparent lamellae structure exhibiting a phyllitic texture. The layered surfaces are uneven, with numerous micro-pores and micro-cracks of varying sizes and irregular shapes. Typically, the pores are more developed, and the number of cracks exceeds the number of pores, leading to poor connectivity between the rock matrix. As a result, phyllite exhibits significant anisotropy in its mechanical properties, showing better toughness and shear strength along the layering direction. At the same time, it is relatively weaker perpendicular to the layering direction.

#### Velocity testing.

**Fig 4:**
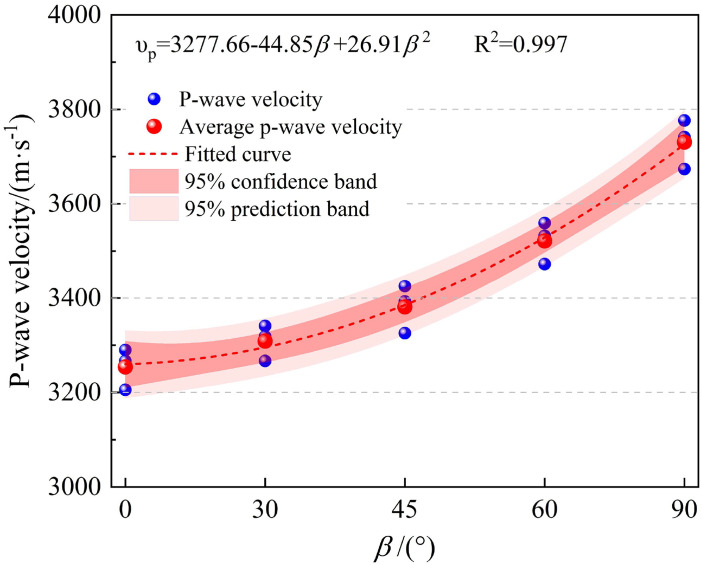
Longitudinal wave velocity characteristics of phyllite specimens at different bedding angles.

Fig 4 shows the variation of P-wave velocity of phyllite specimens at different bedding angles. As seen from the figure, the P-wave velocity of phyllite increases significantly with the increase in bedding angle, indicating that the layered structure significantly affects the propagation velocity of the acoustic waves. When the bedding plane is at 0°, the P-wave velocity is the slowest, while at 90°, the P-wave velocity is the fastest. This is primarily because the bedding planes of the rock exhibit clear orientation, causing different resistance and scattering as the waves propagate. When the propagation direction of the P-wave is parallel to the bedding planes, the wave velocity is the fastest, as the wave encounters less resistance and experiences minimal energy loss. However, when the propagation direction forms a larger angle with the bedding planes, the wave velocity decreases as the waves undergo more reflections and refractions between the bedding planes, resulting in energy attenuation.

The fitting equation between the longitudinal wave velocity and bedding angle is: $v_{p}=3276.82-45.49\beta +26.61\beta^{2}R^{\text{2}}=0\mathrm{.998}$; This quadratic polynomial relationship indicates a nonlinear relationship between the longitudinal wave velocity and bedding angle. The fitted curve explains the data well, meaning the fitting equation accurately describes the trend of longitudinal wave velocity changes with bedding angle.

### Mechanical properties of layered phyllite

#### Stress–strain curves.

**Fig 5:**
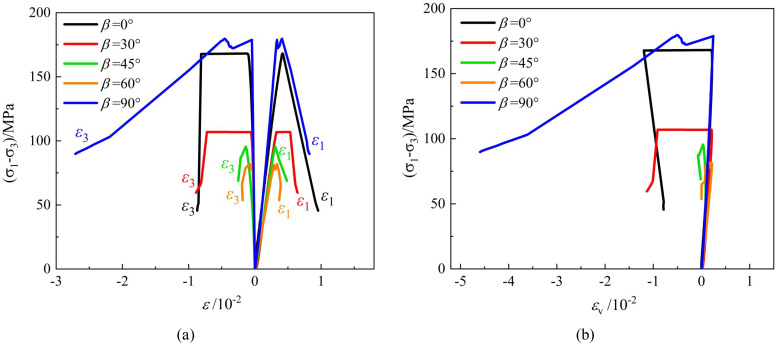
Stress-strain output of triaxial compression tests carried out of layered phyllite at a confining pressure of 10 MPa (a) *ε*_1_ VS *σ*_1_-*σ*_3_, *ε*_3_ VS *σ*_1_-*σ*_3_ (b) *ε*_v_ VS *σ*_1_-*σ*_3_.

**Fig 6:**
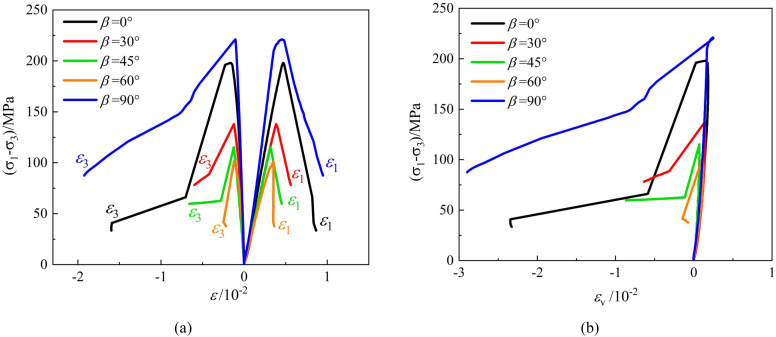
Stress-strain output of triaxial compression tests carried out of layered phyllite at a confining pressure of 20 MPa (a) *ε*_1_ VS *σ*_1_-*σ*_3_, *ε*_3_ VS *σ*_1_-*σ*_3_ (b) *ε*_v_ VS *σ*_1_-*σ*_3_.

**Fig 7:**
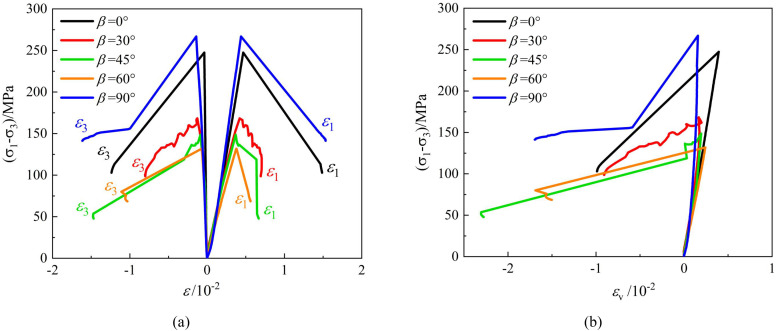
Stress-strain output of triaxial compression tests carried out of layered phyllite at a confining pressure of 30 MPa (a) *ε*_1_ VS *σ*_1_-*σ*_3_, *ε*_3_ VS *σ*_1_-*σ*_3_ (b) *ε*_v_ VS *σ*_1_-*σ*_3_.

The typical deviatoric stress-strain curves of phyllite specimens under conventional triaxial compression at different confining pressures are shown in Figs 5–7. The following observations can be made:

Under different confining pressure conditions, the stress-strain curves of layered phyllite specimens with different bedding angles exhibit similar trends. They all experience stages of pore closure, elastic deformation, plastic yield, and post-peak failure. As the bedding angle increases, the pore closure phase gradually shortens, the slope of the stress-strain curves increases, and the corresponding elastic modulus increases. Near the peak stress, some stress-strain curves show serrated fluctuations, primarily due to localized shear sliding failure along the bedding planes during the loading process. This results in local fluctuations in the stress-strain curve.

At the peak stress, the stress-strain curve drops sharply, and the peak point shows a distinct sharp point feature on the curve. Even under high confining pressures, the stress reduction process remains rapid. This is mainly because, at the moment of failure, the elastic strain energy accumulated inside the specimen is quickly released, causing a sharp decrease in stress and displaying an apparent brittle failure characteristic.

The dilation behavior of layered phyllite is closely related to the bedding angle and confining pressure conditions. Generally, under 10 MPa and 20 MPa confining pressures, phyllite with bedding angles of 0° and 90° exhibits significant lateral and small axial deformation, with stronger dilation behavior. However, the dilation behavior is relatively weak when the bedding angles are 30°, 45°, and 60°. Furthermore, at a confining pressure of 30 MPa, the influence of the bedding angle on the dilation behavior of phyllite diminishes. The main reason for these phenomena is as follows: when the bedding angle is 0°, the bedding planes are parallel to the loading direction, and the confinement restricts the extension of cracks along the bedding planes, leading the rock to show higher compressive strength and minor axial deformation. When the bedding angle is 90°, the bedding planes are perpendicular to the loading direction, and the confinement restricts axial deformation, causing the rock to undergo more lateral expansion, exhibiting more considerable lateral deformation and minor axial deformation. When the bedding angles are 30°, 45°, and 60°, the specimen is more prone to shear sliding failure along the bedding planes in these directions, and the restricting effect of the structural planes on the deformation is enhanced, leading to minor lateral deformation and weaker dilation behavior.

As the confining pressure increases, the external confinement reduces lateral deformation in the phyllite and weakens the effect of bedding planes on the deformation.

#### Strength and deformation parameters.

**Fig 8:**
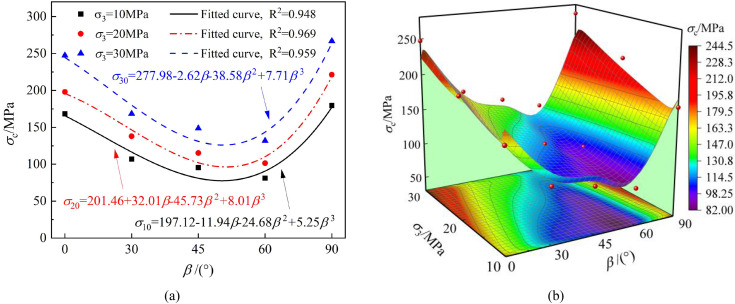
Shows the relationship between peak strength and bedding angle, as well as confining pressure for the phyllite specimens. (a) The effect of bedding angle (b) The combined effect of bedding angle and confining pressure.

**Fig 9:**
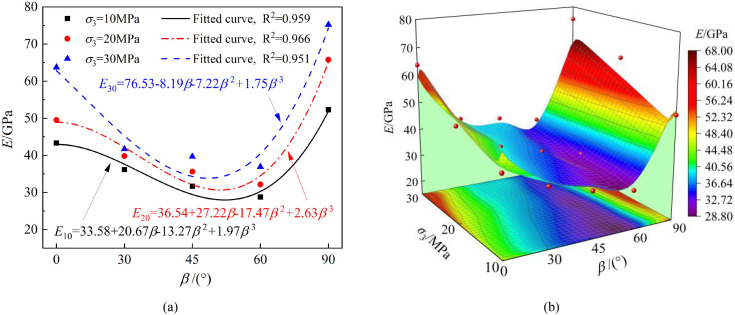
Shows the relationship between the elastic modulus and bedding angle, as well as confining pressure for the phyllite specimens. (a) The effect of bedding angle (b) The combined effect of bedding angle and confining pressure.

**Fig 10:**
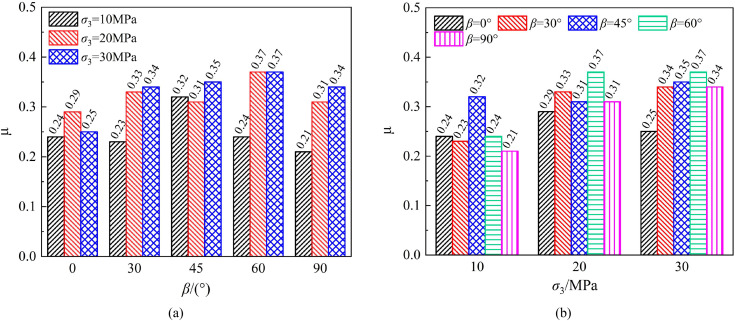
Variation of Poisson’s ratio of phyllite specimens with bedding angle and confining pressure: (a) Effect of bedding angle (b) Effect of confining pressure.

**Fig 11:**
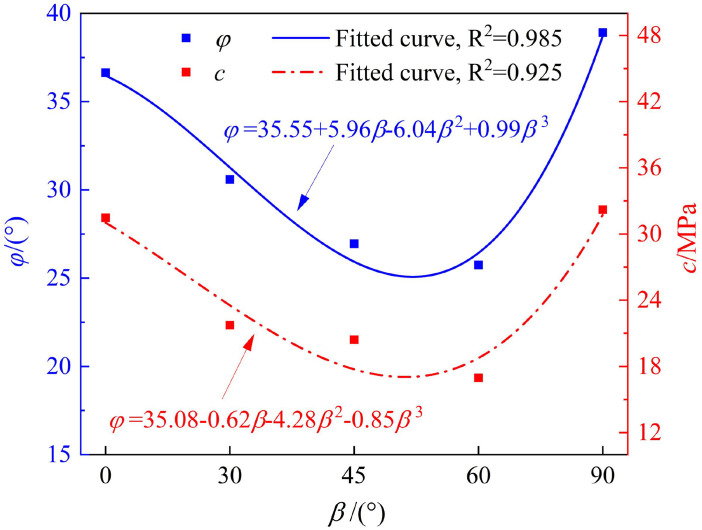
Curves of internal friction angle and cohesion of phyllite specimens at different bedding angles.

The peak strength, elastic modulus, and Poisson’s ratio of the specimens under different bedding angles and confining pressures are shown in Figs 8–11, respectively.

From Fig 8, it can be observed that the peak strength of the phyllite specimens under different confining pressures exhibits a similar trend. The peak strength first decreases and then increases with the increase in bedding angle, showing a “U”-shaped variation. The peak strength is lower at bedding angles of 45° and 60° and higher at bedding angles of 0° and 90°—furthermore, the peak strength increases with increased confining pressure for specimens with the same bedding angle. A cubic function can describe the relationship between peak strength and bedding angle under different confining pressures.

In the triaxial compression test of phyllite specimens, the failure is most likely to occur when the bedding angle is 45° and 60°. The primary reason for this is the weak cementation characteristic of its structural planes. At these specific angles, the failure mode of the phyllite tends to be shear failure along the bedding planes, which is typically accompanied by shear sliding along multiple bedding planes and the propagation of cracks.

Fig 9 shows that the variation in the elastic modulus of the phyllite specimens under different bedding angles follows a similar trend to that of peak strength. Under the same loading conditions, both peak strength and elastic modulus first decrease and then increase as the bedding angle increases, presenting a “U”-shaped variation. This indicates significant anisotropic mechanical properties, which Duan et al. [44] also observed.

Therefore, the variation in bedding angle significantly impacts the bearing capacity of phyllite, which in turn affects the severity of surrounding rock failure. For underground projects under different bedding angles, it is essential to design appropriate support methods based on the bearing capacity of the surrounding rock.

As shown in Fig 10(a), under different confining pressure conditions, the Poisson’s ratio of layered phyllite exhibits a fluctuating trend with changes in bedding angle. However, the differences are relatively small, indicating that the bedding angle has a minor effect on phyllite specimens’ axial and circumferential deformation. Fig 10(b) shows that, under the influence of confining pressure, the Poisson’s ratio of phyllite specimens generally increases with increasing confining pressure at different bedding angles. This suggests that under higher confining pressure conditions, the deformation of layered phyllite becomes more uniform.

Fig 11 shows that the internal friction angle (*φ*) and cohesion (*c*) first decrease and then increase with increasing bedding angle, following a U-shaped distribution, demonstrating the same variation pattern. Specifically, at bedding angles of 0° and 90°, both the internal friction angle and cohesion are relatively high, whereas at bedding angles of 45° and 60°, both parameters are smaller. For example, at bedding angles of 60° and 90°, the internal friction angles of the phyllite specimens are 25.75° and 38.91°, respectively, differing by 33.82%, and the cohesions are 16.97 MPa and 32.21 MPa, respectively, with a difference of 47.31%.

The results are consistent with the effect of the structure surface strength, suggesting that when the bedding angle is β = 45° + φ/2, the phyllite specimens are more prone to shear failure along the weak bedding planes. The difference between *σ*_1_ and *σ*_3_ reaches its minimum, and the shear strength parameters are relatively low [45]. Additionally, the relationship between the internal friction angle, cohesion, and bedding angle approximates a cubic function.

#### Failure mode analysis of layered phyllite.

**Fig 12:**
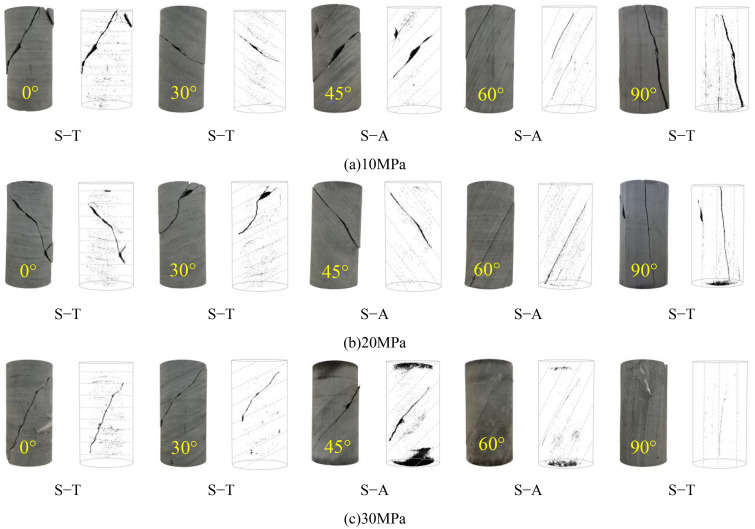
Failure modes of typical phyllite specimens under different bedding angles and confining pressures. (a) 10 MPa, (b) 20 MPa, (c) 30 MPa.

Fig 12 shows the failure modes of phyllite specimens with different layering angles under different confining pressures. The failure modes of phyllite specimens with different layering angles exhibit significant confining pressure and layering effects. The failure modes can be mainly categorized into two types: shear failure through the layering plane (S-T) and shear failure along the layering plane (S-A), which is consistent with the findings of Tien et al. [8]. The two failure modes are analyzed as follows:

(1) Shear Failure Through the Layering Plane (S-T): S-T is the most common type of failure. This failure mode mainly involves shear sliding failure that traverses the matrix body and layering planes. The matrix and layering planes are the primary controlling factors. The shear fracture plane extends along the diagonal of the specimen, passing through most of the layering planes. This type of failure is characterized by tensile cracking.
(2) Shear Failure Along the Layering Plane (S-A): In this failure mode, shear sliding failure mainly occurs along the layering planes. The number and shape of the cracks are usually elementary, with only one or two straight cracks extending along the layering plane. The primary controlling factor is the layering, and due to the weak mechanical properties of the layering planes, S-A failure is more likely to occur when the layering angle is β = 45°–60°. The layering effect significantly induces the failure mode of layered phyllite. Additionally, high confining pressure suppresses the layering effect of layered phyllite. For example, at a confining pressure of 10 MPa, phyllite specimens with layering angles of 45° and 60° produced two macro shear planes, whereas at confining pressures of 20 MPa and 30 MPa, phyllite specimens with layering angles of 45° and 60° produced only one macro shear plane.

## Energy evolution characteristics of layered phyllite

### Law of conservation of energy

During rock mass deformation and failure process, they continuously exchange material and energy with the surrounding environment in various forms. According to the first law of thermodynamics, assuming no heat exchange occurs between the rock and the surrounding environment during the experiment, the mechanical energy input during the deformation of the rock will only be converted into elastic strain energy within the rock, and the energy dissipated during the rock’s damage process [46]:


$$U=U^{e}+U^{d}$$ (1)

Where *U* represents the total energy absorbed by the rock mass (kJ/m³), *U*^e^ represents the elastic strain energy (kJ/m³), and *U*^d^ represents the dissipative energy (kJ/m³).

In the principal stress space, the energy components in the rock mass element are:


$$U=\int_{0}^{\varepsilon_{1}}\sigma_{1}d\varepsilon_{1}+\int_{0}^{\varepsilon_{2}}\sigma_{2}d\varepsilon_{2}+\int_{0}^{\varepsilon_{3}}\sigma_{3}d\varepsilon_{3}$$ (2)

$$U^{e}=\frac{\text{1}}{\text{2}}\sigma_{1}\varepsilon_{{}_{1}}^{e}+\frac{\text{1}}{\text{2}}\sigma_{2}\varepsilon_{{}_{2}}^{e}+\frac{\text{1}}{\text{2}}\sigma_{3}\varepsilon_{{}_{3}}^{e}$$ (3)

Where *σ*_1_, *σ*_2_, *σ*_3_ denote the principal stresses in the three directions, *ε*_1_, *ε*_2_, *ε*_3_ denote the corresponding strains in the three directions, and *ε*_1_^e^, *ε*_2_^e^, *ε*_3_^e^ denote the corresponding elastic strains in the three directions.

According to Hooke’s law, the relationship between the principal stresses and the principal strains is:


$$\left\{ \begin{array}{c} \varepsilon_{{}_{1}}^{e}=\frac{\text{1}}{E_{0}}\left[\sigma_{1}-\mu \left(\sigma_{2}+\sigma_{3}\right)\right]\\ \varepsilon_{{}_{2}}^{e}=\frac{\text{1}}{E_{0}}\left[\sigma_{2}-\mu \left(\sigma_{1}+\sigma_{3}\right)\right]\\ \varepsilon_{{}_{3}}^{e}=\frac{\text{1}}{E_{0}}\left[\sigma_{3}-\mu \left(\sigma_{1}+\sigma_{2}\right)\right] \end{array}\right.$$ (4)

Where *E* represents the rock’s elastic modulus, and μ represents the Poisson’s ratio.

By substituting Equation (4) into Equation (3), we get:


$$U^{e}=\frac{\text{1}}{2E}\left[{\sigma_{1}}^{2}+{\sigma_{2}}^{2}+{\sigma_{3}}^{2}-2\mu \left(\sigma_{1}\sigma_{2}+\sigma_{2}\sigma_{3}+\sigma_{1}\sigma_{3}\right)\right]$$ (5)

Where *E*_0_ represents the unloading elastic modulus, which can be simplified to the initial elastic modulus *E*, the slope of the linear segment of the stress-strain curve, and *μ* represents Poisson’s ratio.

The axial pressure works positively on the specimen in a conventional triaxial compression test (*σ*_2_* = σ*_3_). When axial compression causes the specimen to undergo lateral expansion, the confining pressure transitions from doing positive work on the specimen to doing negative work. Therefore, the total energy absorbed by the rock mass U under conventional triaxial compression test conditions can be expressed as:


$$U=U_{1}+U_{3}$$ (6)

Where *U*_1_ represents the strain energy produced by the axial stress *σ*_1_ compression, and *U*_3_ represents the strain energy produced by the confining pressure *σ*_3_.

At any moment *t* during the conventional triaxial compression test, the elastic strain energy produced by the axial stress and confining pressure is:


$$\left\{ \begin{array}{c} U_{1}=\int_{0}^{\varepsilon_{1t}}\sigma_{1}d\varepsilon_{1}\\ U_{3}=2\int_{0}^{\varepsilon_{3t}}\sigma_{3}d\varepsilon_{3} \end{array}\right.$$ (7)

Where ε_1t_ and *ε*_*3t*_ denote the specimen’s axial strain and radial strain at time t during the test, respectively.

According to the basic concept of calculus, the strain energy generated by axial stress and confining pressure can be obtained by summing the areas of small rectangles [47]. The terms *U*_1_ and *U*_3_ in Equation (7) can be calculated as follows:


$$\left\{ \begin{array}{c} U_{1}=\sum_{i=0}^{n}\frac{\text{1}}{\text{2}}\left(\varepsilon_{1}^{i+1}-\varepsilon_{1}^{i}\right)\left(\sigma_{1}^{i+1}+\sigma_{1}^{i}\right)\\ U_{3}=\sum_{i=0}^{n}\left(\varepsilon_{3}^{i+1}-\varepsilon_{3}^{i}\right)\left(\sigma_{3}^{i+1}+\sigma_{3}^{i}\right) \end{array}\right.$$ (8)

Where *σ*_1i_, *σ*_1i−1_, *ε*_1i_, and *ε*_1i−1_ denote the stress and strain values corresponding to any two adjacent points on the stress-strain curve, and *n* denote the total number of sampling points on the stress-strain curve.

By combining Equations (1), (5), and (6), the total energy, elastic energy, and dissipated energy of the rock under conventional triaxial compression test conditions can be obtained as follows:


$$U=U_{1}+U_{3}=\int_{0}^{\varepsilon_{1}}\sigma_{1}d\varepsilon_{1}+2\int_{0}^{\varepsilon_{3}}\sigma_{3}d\varepsilon_{3}$$ (9)

$$U^{e}=\frac{\text{1}}{2E}\left[\sigma_{1}^{2}+2\sigma_{3}^{2}-2\mu \left(2\sigma_{1}\sigma_{3}+\sigma_{3}^{2}\right)\right]$$ (10)

$$U^{d}=U_{1}+U_{3}-U^{e}$$ (11)

### Energy evolution curve

**Fig 13:**
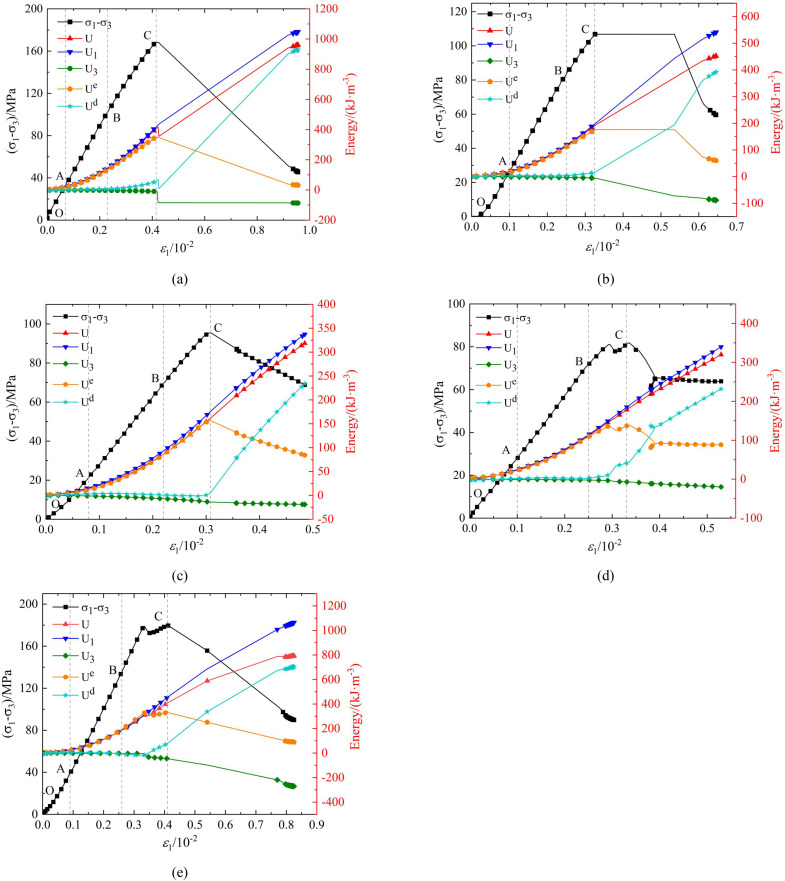
Strain energy and axial strain relationship curves of phyllite specimens with different bedding angles under 10 MPa confining pressure (a) *β *= 0°(b) *β* = 30°(c) *β* = 45°(d) *β* = 60°(e) *β* = 90°.

**Fig 14:**
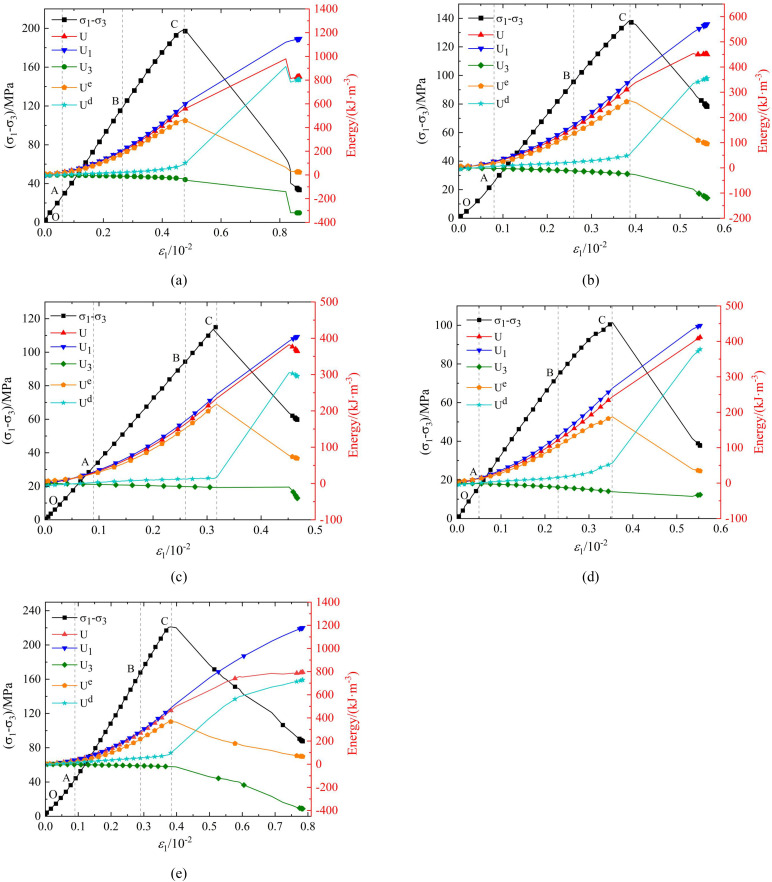
Strain energy and axial strain relationship curves of phyllite specimens with different bedding angles under 20 MPa confining pressure (a) *β* = 0°(b) *β* = 30°(c) *β* = 45°(d) *β* = 60°(e) *β* = 90°.

**Fig 15:**
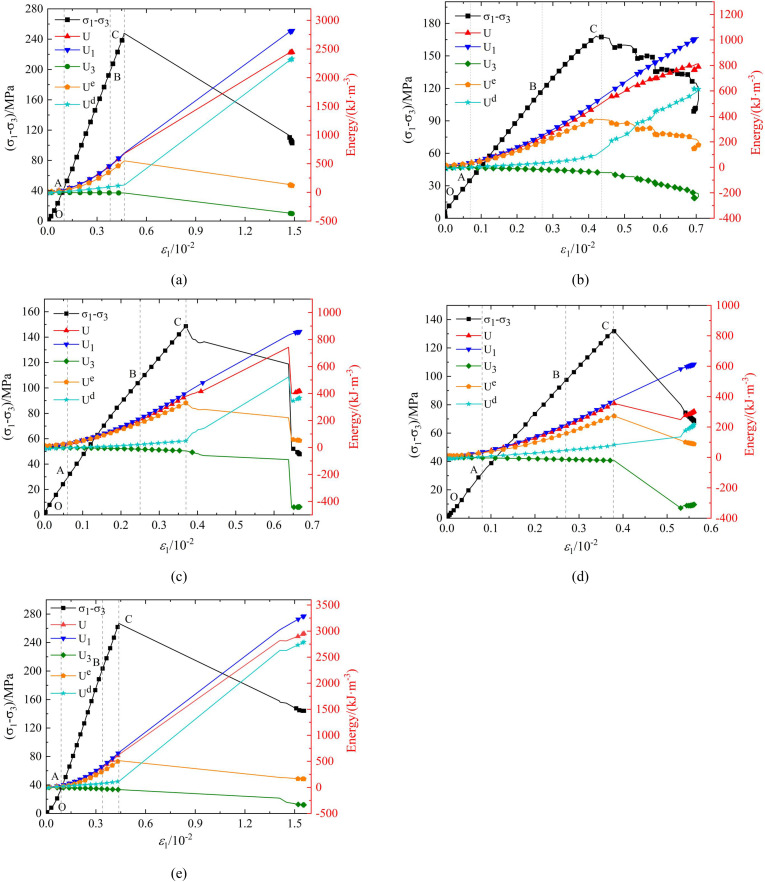
Strain energy and axial strain relationship curves of phyllite specimens with different bedding angles under 30 MPa confining pressure *β* = 0°(b) *β* = 30°(c) *β* = 45°(d) *β* = 60°(e) *β* = 90°.

Figs 13–15 show the strain energy and axial strain relationship curves for phyllite specimens with different bedding angles under various confining pressures. The figures show that, under a given confining pressure, the total absorption energy (*U*) and the energy input by axial pressure (*U*_1_) exhibit similar trends, positively correlating with axial strain and increasing as axial strain increases. The strain energy dissipated due to the confining pressure (U_3_) also increases with axial strain. The elastic strain energy (*U*^e^) changes similarly to the stress-strain curve, first increasing and decreasing as axial strain increases. In contrast, the dissipated energy (U^d^) shows different stages of change throughout the deformation and failure process, including steady, nonlinear, and rapid increases.

The strain energy density curves under different confining pressure conditions can be divided into four stages:

Porosity and Fracture Compaction Stage (OA Section): During this stage, the pores and fractures inside the specimen gradually close as the load is applied, and the rock is compacted. The energy input from axial pressure (U_1_), increases non-linearly at a relatively slow rate. The growth rates of elastic strain energy (*U*^e^), and dissipated energy (*U*^d^), are almost the same initially. Subsequently, the growth rate of elastic strain energy (*U*^e^), gradually increases while the growth rate of dissipated energy (*U*^d^), decreases. The difference in the growth rates of the two energies increases as the deformation progresses. During this process, the phyllite specimens experience slight expansion deformation, and the strain energy (*U*_3_), consumed by the confining pressure doing negative work on the sample is minimal, nearly zero. This stage ends when the pores and fractures close, and the specimen enters the elastic deformation phase.

Elastic Deformation Stage (AB segment): In this stage, the phyllite specimens undergoe further compaction, and the pores and fractures are fully closed. During loading, there is no significant plastic deformation or damage, so the energy input from the axial pressure (*U*_1_) and the elastic strain energy (*U*^e^) continue to increase, following an approximately linear growth trend. Meanwhile, the dissipated energy (*U*^d^) remains almost constant. The phyllite specimens begin to experience lateral deformation, leading to a slow increase in strain energy (*U*_3_) consumed by the confining pressure. If unloading occurs in this stage, the accumulated elastic strain energy (*U*^e^) can be fully released along the original loading path.

Plastic Yield Stage (BC segment): As the deformation increases, the sample enters the plastic deformation stage. The energy input from the axial pressure (*U*_1_) continues to increase, but the rate of increase slows down. The internal development, nucleation, and coalescence of microcracks lead to intensified plastic deformation, reducing the portion of elastic deformation and causing an increase in the growth rate for dissipated energy (*U*^d^). The rate of increase in elastic strain energy (*U*^e^) also slows down. At the same time, the phyllite specimens’ lateral deformation continues to increase, which leads to a growing rate of strain energy (*U*_3_) consumed by the confining pressure.

Post-Peak Failure Stage (After point C): The sample exhibits macro-cracks in this stage, and both axial and lateral deformations intensify. The energy input from the axial pressure (*U*_1_) rapidly increases, and the growth rate of the strain energy consumed by the confining pressure (*U*_3_) also increases. The formation and propagation of macro-cracks result in a rapid release of the accumulated elastic strain energy (*U*^e^), causing a sharp decrease (*U*^e^) and a rapid increase in dissipated energy (*U*^d^). The rapid release of energy accelerates cracks’ propagation and circumferential expansion, ultimately leading to sample instability and failure.

Under the same confining pressure (*U*_3_), the energy indicators of phyllite specimens with different bedding angles (total absorption energy (*U*), elastic strain energy (*U*^e^), and dissipated energy (*U*^d^)) are different. The total absorption energy (*U*), elastic strain energy (*U*^e^), and dissipated energy (*U*^d^) are relatively higher for the phyllite specimens with lamination angles of 0° and 90°. In comparison, the phyllite specimens with lamination angles of 30°, 45°, and 60° have the lowest energy accumulation and release efficiency.

Under the same bedding angle, the energy indicators (total energy absorbed by the rock (*U*), elastic strain energy (*U*^e^), and dissipated energy (*U*^d^)) of phyllite specimens with different confining pressures (*U*_3_) are also different. With an increase in confining pressure (*U*_3_), the total absorption energy (*U*) and elastic strain energy (*U*^e^) increase significantly. These phenomena reflect the complexity of energy distribution during deformation and failure processes and highlight the significant influence of bedding angle and confining pressure on the rock’s mechanical behavior and energy evolution.

### Strain energy evolution anisotropy characteristics

As observed in Section 4.2, during the entire loading process, the total absorption energy (*U*), elastic strain energy (*U*^e^), and dissipated energy (*U*^d^) absorbed by the layered phyllite specimens are different. To further study the influence of bedding angle on the energy evolution of phyllite, the relationship between strain energy at the peak stress point and bedding angle under triaxial compression conditions was analyzed. The strain energy at the peak stress point is defined as peak total absorption energy, peak elastic strain energy, and peak dissipated energy, represented as *U*_p_, *U*_p_^e^, and *U*_p_^d^, respectively.

**Fig 16:**
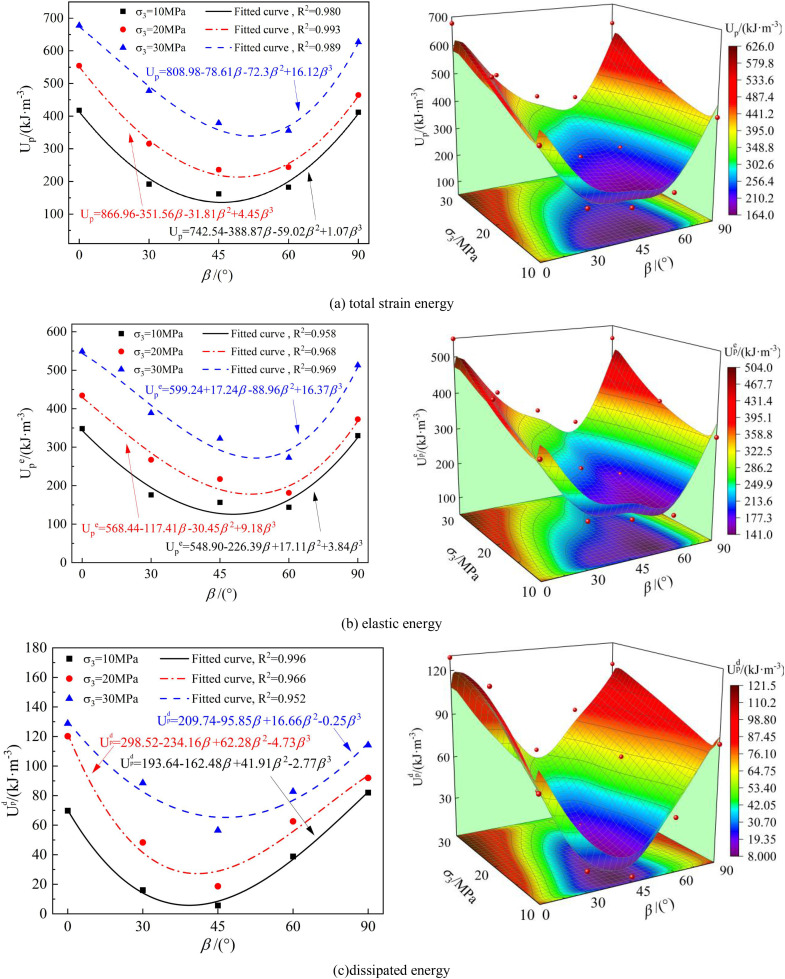
Variation of strain energy at peak stress point with layering angle and confining pressure. (a) total strain energy, (b) elastic energy, (c) dissipated energy.

The phyllite specimens’ peak total absorption energy (*U*_p_), peak elastic strain energy (*U*_p_^e^), and peak dissipated energy (*U*_p_^d^) were fitted, and the resulting relationships with the bedding angle are shown in Fig 16.

The fitting equations for these relationships are as follows:


$$J=A+B_{1}\cdot \beta^{1}+B_{2}\cdot \beta^{2}+B_{3}\cdot \beta^{3}$$

In the formula, J represents the energy indicators (*U*_p,_ *U*_p_^e^, and *U*_p_^d^), A is the intercept of the fitting expression, B_1_, B_2_, and B_3_ are the coefficients of the fitting expression, and β is the bedding angle. The fitting correlation coefficient R^2^ ranges from 0.958 to 0.993, indicating a good fitting result.

As shown in Fig 16, under different confining pressures, the distribution patterns of peak total absorption energy (*U*_p_), peak elastic strain energy (*U*_p_^e^), and peak dissipated energy (*U*_p_^d^) for phyllite specimens at different bedding angles also follow a “U” shape distribution, exhibiting significant anisotropy, which is consistent with previous studies [48,49]. The phyllite specimens with bedding angles of 45° and 60° require the least total energy for failure and are more prone to failure, while the specimens with bedding angles of 0° and 90° require the total energy for failure and are less likely to fail. The primary reason for this phenomenon is that the bedding planes are the weak planes in layered phyllite, with their strength and deformation characteristics significantly lower than the matrix. The difference in bedding angle leads to varying stress states of the bedding planes during the loading process. When the bedding angle is 0° or 90°, the weak plane effect is suppressed, and failure typically occurs through shear failure involving both the rock matrix and bedding, resulting in more energy loss. When the bedding angle is 45° or 60°, failure mainly involves the inclined sliding friction of the interlayer structure, resulting in less energy loss.

It can also be seen that the energy evolution characteristics of phyllite specimens show a confining pressure effect, with an increase in confining pressure significantly increasing the total absorption energy (*U*_p_), peak elastic strain energy (*U*_p_^e^), and peak dissipated energy (*U*_p_^d^). For example, when the confining pressure increases from 10 MPa to 20 MPa, the total absorption energy (*U*_p_), peak elastic strain energy (*U*_p_^e^), and peak dissipated energy (*U*_p_^d^) of the phyllite specimens at a bedding angle of 0° increase by 32.61%, 24.67%, and 72.19%, respectively. When the confining pressure increases from 20 MPa to 30 MPa, the total absorption energy (*U*_p_), peak elastic strain energy (*U*_p_^e^) of the phyllite specimens at a bedding angle of 0° increase by 22.22%, 26.39%, and 7.16%, respectively. The reason is that the increase in confining pressure (*U*_3_) restricts the lateral deformation of the rock, suppresses the expansion of internal microcracks, and inhibits shear sliding along the bedding planes, enhancing the overall integrity and mechanical performance of the rock, and the elastic deformation stage of the rock is prolonged, allowing more energy to be stored within the rock.

In underground space engineering (such as roadways and tunnels), when the direction of the maximum principal stress forms an angle of approximately 45° to 60° with the bedding planes of the layered rock mass, the elastic strain energy stored in the rock mass is most likely to reach its limit and be rapidly released along the bedding planes. This release of energy may lead to instability of the surrounding rock, potentially triggering dynamic disasters such as rockbursts. Therefore, in engineering design and construction, it is essential to pay close attention to the stability of the surrounding rock in layered rock masses, optimize support schemes, and implement appropriate measures to ensure the project’s safety.

In underground engineering projects (such as roadways and tunnels), when the direction of the maximum principal stress forms an angle of approximately 45° to 60° with the bedding planes of the layered rock mass, the elastic strain energy stored in the rock mass is more likely to reach its critical value and be rapidly released along the layering plane. This release of energy may lead to rock mass instability and even trigger dynamic disasters such as rock bursts. Therefore, in practical underground engineering design and construction, it is essential to consider the angle between the maximum principal stress direction and the orientation of the layering in the rock mass and to design support measures accordingly to prevent and control such disasters effectively.

## Conclusions

This study focuses on the layered phyllite of the Shenzuo Tunnel along the Jiuma Expressway. Conventional triaxial compression tests were conducted on layered phyllite specimens with different bedding angles (0°, 30°, 45°, 60°, and 90°) to investigate their mechanical behavior, deformation parameters, and failure modes. Additionally, based on the principle of energy conservation, the energy evolution characteristics of the layered phyllite were explored. The main conclusions are as follows:

(1) The stress-strain curves of layered phyllite under different confining pressures and bedding angles go through four stages: pore and fissure compression, elastic deformation, plastic yielding, and post-peak failure, exhibiting an overall brittle failure characteristic. The bedding angle and confining pressure significantly impact the dilation behavior. The effect of bedding angle is more pronounced at confining pressures of 10 MPa and 20 MPa, especially at bedding angles of 0° and 90°. However, at a confining pressure of 30 MPa, the influence of bedding angle on dilation behavior becomes less significant.
(2) As the bedding angle increases from 0° to 90°, the peak strength, elastic modulus, internal friction angle, and cohesion of the layered phyllite show a trend of first decreasing and then increasing, reaching a minimum at bedding angles of 45° and 60°, and relatively higher values at 0° and 90°. The anisotropy of the Poisson’s ratio is not significant. With the increase in confining pressure, the mechanical properties of the rock improve, and the anisotropic characteristics gradually weaken.
(3) The failure modes of the layered phyllite are mainly categorized into shear failure across the bedding plane (S-T) and shear failure along the bedding plane (S-A). The bedding angle and confining pressure significantly influence the failure mode. At 45° and 60° bedding angles, shear slip failure along the bedding plane is more common. High confining pressure suppresses the weak plane effect of the bedding, reduces the number of macro shear planes, and enhances the integrity and stability of the rock.
(4) The total absorption energy (*U*) and dissipated energy (*U*^d^) by the phyllite with different bedding angles increase with axial strain. The elastic strain energy (*U*^e^) increases first with axial strain and then decreases. The peak absorption total energy absorbed (U_p_), peak elastic strain energy (*U*_p_^e^), and peak dissipated energy (*U*_p_^d^) all exhibit a “U”-shaped distribution, with higher values at bedding angles of 0° and 90° and lower values at 45° and 60°, showing significant anisotropy. Additionally, these values increase nonlinearly with the increase in confining pressure.

## References

[1] Naumann M, Hunsche U, Schulze O. Experimental investigations on anisotropy in dilatancy, failure and creep of Opalinus Clay. Phys Chem Earth Parts A/B/C. 2007;32(8–14):889–95. doi: 10.1016/j.pce.2005.04.006

[2] Wong LNY, Einstein HH. Systematic evaluation of cracking behavior in specimens containing single flaws under uniaxial compression. Int J Rock Mech Min Sci. 2009;46(2):239–49. doi: 10.1016/j.ijrmms.2008.03.006

[3] Yin Q, Liu R, Jing H, Su H, Yu L, He L. Experimental study of nonlinear flow behaviors through fractured rock samples after high-temperature exposure. Rock Mech Rock Eng. 2019;52(9):2963–83. doi: 10.1007/s00603-019-1741-0

[4] Man J, Zhou M, Zhang D, Huang H, Chen J. Face stability analysis of circular tunnels in layered rock masses using the upper bound theorem. J Rock Mech Geotech Eng. 2022;14(6):1836–48. doi: 10.1016/j.jrmge.2021.12.023

[5] Liu M, Luo X, Bi R, Zhou J, Du K. Impacts of bedding angle and cementation type of bedding planes on mechanical behavior of thin-layer structured bedded rocks under uniaxial compression. Geomech Energy Environ. 2023;35:100473. doi: 10.1016/j.gete.2023.100473

[6] Ma Q, Liu X, Tan Y, Elsworth D, Shang J, Song D, et al. Numerical study of mechanical properties and microcrack evolution of double-layer composite rock specimens with fissures under uniaxial compression. Eng Fract Mech. 2023;289:109403. doi: 10.1016/j.engfracmech.2023.109403

[7] Ajalloeian R, Lashkaripour GR. Strength anisotropies in mudrocks. Bull Eng Geol Environ. 2000;59(3):195–9. doi: 10.1007/s100640000055

[8] Tien YM, Kuo MC, Juang CH. An experimental investigation of the failure mechanism of simulated transversely isotropic rocks. Int J Rock Mech Min Sci. 2006;43(8):1163–81. doi: 10.1016/j.ijrmms.2006.03.011

[9] Bonini M, Debernardi D, Barla M, Barla G. The mechanical behaviour of clay shales and implications on the design of tunnels. Rock Mech Rock Eng. 2007;42(2):361–88. doi: 10.1007/s00603-007-0147-6

[10] Cho J-W, Kim H, Jeon S, Min K-B. Deformation and strength anisotropy of Asan gneiss, Boryeong shale, and Yeoncheon schist. Int J Rock Mech Min Sci. 2012;50:158–69. doi: 10.1016/j.ijrmms.2011.12.004

[11] Goricki A, Pimentel E. Triaxial tests on cataclasites. Rock Mech Rock Eng. 2014;48(5):2167–71. doi: 10.1007/s00603-014-0668-8

[12] Geng Z, Chen M, Jin Y, Yang S, Yi Z, Fang X, et al. Experimental study of brittleness anisotropy of shale in triaxial compression. J Nat Gas Sci Eng. 2016;36:510–8. doi: 10.1016/j.jngse.2016.10.059

[13] Feng G, Kang Y, Wang X, Hu Y, Li X. Investigation on the failure characteristics and fracture classification of shale under brazilian test conditions. Rock Mech Rock Eng. 2020;53(7):3325–40. doi: 10.1007/s00603-020-02110-6

[14] Peng Y, Du Z, Chen P, Yao Y, Liu G, Wu L. Study on dynamic mechanical properties and failure pattern of thin-layered schist. Appl Sci. 2024;14(19):9101. doi: 10.3390/app14199101

[15] Fereidooni D, Khanlari GR, Heidari M, Sepahigero AA, Kolahi-Azar AP. Assessment of inherent anisotropy and confining pressure influences on mechanical behavior of anisotropic foliated rocks under triaxial compression. Rock Mech Rock Eng. 2016;49(6):2155–63. doi: 10.1007/s00603-015-0814-y

[16] Tavallali A, Vervoort A. Effect of layer orientation on the failure of layered sandstone under Brazilian test conditions. Int J Rock Mech Min Sci. 2010;47(2):313–22. doi: 10.1016/j.ijrmms.2010.01.001

[17] Li D, Wong LNY, Liu G, Zhang X. Influence of water content and anisotropy on the strength and deformability of low porosity meta-sedimentary rocks under triaxial compression. Eng Geol. 2012;126:46–66. doi: 10.1016/j.enggeo.2011.12.009

[18] Kim H, Cho J-W, Song I, Min K-B. Anisotropy of elastic moduli, P-wave velocities, and thermal conductivities of Asan Gneiss, Boryeong Shale, and Yeoncheon Schist in Korea. Engineering Geology. 2012;147–148:68–77. doi: 10.1016/j.enggeo.2012.07.015

[19] Guo Y, Li X, Huang L, Dyskin A, Pasternak E. Insight into the dynamic tensile behavior of deep anisotropic shale reservoir after water-based working fluid cooling. Int J Rock Mech Min Sci. 2024;182:105875. doi: 10.1016/j.ijrmms.2024.105875

[20] Song T, Feng X-T, Zhou Y, Yang C, Yu X. Anisotropic characteristics and creep model for thin-layered rock under true triaxial compression. J Rock Mech Geotech Eng. 2024;16(12):4815–34. doi: 10.1016/j.jrmge.2024.02.018

[21] Behrestaghi MHN, Seshagiri Rao K, Ramamurthy T. Engineering geological and geotechnical responses of schistose rocks from dam project areas in India. Eng Geol. 1996;44(1–4):183–201. doi: 10.1016/s0013-7952(96)00069-5

[22] Martin McCabe W, Koerner RM. High pressure shear strength investigation of an anisotropic mica schist rock. Int J Rock Mech Min Sci Geomech Abstr. 1975;12(8):219–28. doi: 10.1016/0148-9062(75)91402-3

[23] Chen Z, He C, Xu G, Ma G, Wu D. A case study on the asymmetric deformation characteristics and mechanical behavior of deep-buried tunnel in phyllite. Rock Mech Rock Eng. 2019;52(11):4527–45. doi: 10.1007/s00603-019-01836-2

[24] Liu X, Meng Y, Jing H, Liu W, Wan C, Cao Y, et al. Effects of bedding plane properties on mechanical, acoustic emission and micro failure characteristics of bedded rock mass. Bull Eng Geol Environ. 2024;83(5). doi: 10.1007/s10064-024-03693-y

[25] Li D, Sun Z, Xie T, Li X, Ranjith PG. Energy evolution characteristics of hard rock during triaxial failure with different loading and unloading paths. Eng Geol. 2017;228:270–81. doi: 10.1016/j.enggeo.2017.08.006

[26] Zhang Y, Feng X-T, Yang C, Zhang X, Sharifzadeh M, Wang Z. Fracturing evolution analysis of Beishan granite under true triaxial compression based on acoustic emission and strain energy. Int J Rock Mech Min Sci. 2019;117:150–61. doi: 10.1016/j.ijrmms.2019.03.029

[27] Xie H, Li L, Peng R, Ju Y. Energy analysis and criteria for structural failure of rocks. J Rock Mech Geotech Eng. 2009;1(1):11–20. doi: 10.3724/sp.j.1235.2009.00011

[28] Gong S, Wang Z, Zhou L. Dynamic fracture mechanics and energy distribution rate response characteristics of coal containing bedding structure. PLoS One. 2021;16(6):e0247908. doi: 10.1371/journal.pone.0247908 34166380 PMC8224884

[29] Zhou Y, Su S, Li P. Mechanical behavior, energy release, and crack distribution characteristics of water‐saturated phyllite under triaxial cyclic loading. Adv Civ Eng. 2021;2021(1). doi: 10.1155/2021/3681439

[30] Chang X, Zhang X, Qian LZ, Chen SH, Yu J. Influence of bedding anisotropy on the dynamic fracture behavior of layered phyllite. Eng Fract Mech. 2022;260:108183. doi: 10.1016/j.engfracmech.2021.108183

[31] Zhai M, Li L, Chen B, Zhang Q, Zhang Z, Zhang L. Investigation on the anisotropy of mechanical properties and brittleness characteristics of deep laminated sandstones. Eng Fract Mech. 2023;289:109386. doi: 10.1016/j.engfracmech.2023.109386

[32] Wang C, Li E, Zhang D, Han Y, Lu H, He K, et al. Mechanical properties and energy evolution of Beishan shallow-layer granite under different unloading paths. J Mt Sci. 2024;21(5):1728–44. doi: 10.1007/s11629-023-8591-7

[33] Zhang J, Ai C, Li Y, Che M, Gao R, Zeng J. Energy-based brittleness index and acoustic emission characteristics of anisotropic coal under triaxial stress condition. Rock Mech Rock Eng. 2018;51(11):3343–60. doi: 10.1007/s00603-018-1535-9

[34] Zhang Q, Meng X, Zhao G. Energy evolution and fractal characteristics of sandstones under true triaxial cyclic loading and unloading. Fractal Fract. 2024;8(12):714. doi: 10.3390/fractalfract8120714

[35] Chen M, Liu Z, Wang X, Zheng J, Yang L, Bai F, et al. Investigation of fatigue mechanics and crack evolution characteristics of jointed specimens under cyclic uniaxial compression. Fatigue Fract Eng Mat Struct. 2024;48(1):261–78. doi: 10.1111/ffe.14471

[36] Khanlari G-R, Heidari M, Sepahigero A-A, Fereidooni D. Quantification of strength anisotropy of metamorphic rocks of the Hamedan province, Iran, as determined from cylindrical punch, point load and Brazilian tests. Eng Geol. 2014;169:80–90. doi: 10.1016/j.enggeo.2013.11.014

[37] Heng S, Guo Y, Yang C, Daemen JJK, Li Z. Experimental and theoretical study of the anisotropic properties of shale. Int J Rock Mech Min Sci. 2015;74:58–68. doi: 10.1016/j.ijrmms.2015.01.003

[38] Xu G, He C, Su A, Chen Z. Experimental investigation of the anisotropic mechanical behavior of phyllite under triaxial compression. Int J Rock Mech Min Sci. 2018;104:100–12. doi: 10.1016/j.ijrmms.2018.02.017

[39] Gao M, Liang Z, Jia S, Zhang Q, Zou J. Energy evolution analysis and related failure criterion for layered rocks. Bull Eng Geol Environ. 2023;82(12). doi: 10.1007/s10064-023-03445-4

[40] Fairhurst CE, Hudson JA. Draft ISRM suggested method for the complete stress-strain curve for intact rock in uniaxial compression. Int J Rock Mech Min Sci Geomech Abstr 1999;36:281–9. doi: 10.1016/S0148-9062(99)00006-6

[41] Ersoy H, Karahan M, Kolaylı H, Sünnetci MO. Influence of mineralogical and micro-structural changes on the physical and strength properties of post-thermal-treatment clayey rocks. Rock Mech Rock Eng. 2020;54(2):679–94. doi: 10.1007/s00603-020-02282-1

[42] Wu Y, Huang L, Li X. Dynamic compression behaviors of heat-treated granite under combined dynamic and static load. Bull Eng Geol Environ. 2024;83(5). doi: 10.1007/s10064-024-03664-3

[43] Yan D, Ruan S, Chen S, Liu Y, Tian Y, Wang H, et al. Effects and mechanisms of surfactants on physical properties and microstructures of metakaolin-based geopolymer. J Zhejiang Univ Sci A. 2021;22(2):130–46. doi: 10.1631/jzus.a2000059

[44] Duan M, Jiang C, Yin W, Yang K, Li J, Liu Q. Experimental study on mechanical and damage characteristics of coal under true triaxial cyclic disturbance. Eng Geol. 2021;295:106445. doi: 10.1016/j.enggeo.2021.106445

[45] Zhang L, Niu F, Liu M, Ju X, Wang Z, Wang J, et al. Fracture characteristics and anisotropic strength criterion of bedded sandstone. Front Earth Sci. 2022;10. doi: 10.3389/feart.2022.879332

[46] Peng R, Ju Y, Wang JG, Xie H, Gao F, Mao L. Energy dissipation and release during coal failure under conventional triaxial compression. Rock Mech Rock Eng. 2014;48(2):509–26. doi: 10.1007/s00603-014-0602-0

[47] Li X, Wu Y, Huang L. Mechanical behavior and thermal damage characterization of granite after flame jet-water cooling treatment. Int J Rock Mech Min Sci. 2024;180:105833. doi: 10.1016/j.ijrmms.2024.105833

[48] Duan X, Wang W, Liu S, Cao Y, Zheng Z, Zhu Q. Experimental investigation on mechanical behavior, energy evolution and gas permeability of anisotropic phyllite subjected to triaxial compression and cyclic loading. Geomech Energy Environ. 2023;35:100483. doi: 10.1016/j.gete.2023.100483

[49] Zhang J, Zhang X, Huang Z, Huang J. A brittle-ductile index for transversely isotropic rock based on energy evolution of the characteristic stress and its application. Bull Eng Geol Environ. 2023;82(1). doi: 10.1007/s10064-022-03060-9

